# The Fair Allocation of Scarce Medical Resources: A Comparative Study From Jordan

**DOI:** 10.3389/fmed.2020.603406

**Published:** 2021-01-12

**Authors:** Muhannad H. Yousef, Yazan N. Alhalaseh, Razan Mansour, Hala Sultan, Naseem Alnadi, Ahmad Maswadeh, Yasmeen M. Al-Sheble, Raghda Sinokrot, Khawlah Ammar, Asem Mansour, Maysa Al-Hussaini

**Affiliations:** ^1^School of Medicine, University of Jordan, Amman, Jordan; ^2^Department of Internal Medicine, King Hussein Cancer Center, Amman, Jordan; ^3^Office of Scientific Affairs and Research, King Hussein Cancer Center, Amman, Jordan; ^4^King Hussein Cancer Center, Amman, Jordan; ^5^Human Research Protection Program, King Hussein Cancer Center, Amman, Jordan; ^6^Department of Pathology and Laboratory Medicine, King Hussein Cancer Center, Amman, Jordan

**Keywords:** scarce medical resources, FAIR, COVID-19, Jordan, justice

## Abstract

The allocation strategies during challenging situations among the different social groups is based on 9 principles which can be considered either individually: sickest first, waiting list, prognosis, youngest first, instrumental values, lottery, monetary contribution, reciprocity, and individual behavior, or in combination; youngest first and prognosis, for example. In this study, we aim to look into the most important prioritization principles amongst different groups in the Jordanian population, in order to facilitate the decision-making process for any potential medical crisis. We conducted an online survey that tackled how individuals would deal with three different scenarios of medical scarcity: (1) organ donation, (2) limited hospital beds during an influenza epidemic, and (3) allocation of novel therapeutics for lung cancer. In addition, a free-comment option was included at the end of the survey if respondents wished to contribute further. Seven hundred and fifty-four survey responses were gathered, including 372 males (49.3%), and 382 females (50.7%). Five groups of individuals were represented including religion scholars, physicians, medical students, allied health practitioners, and lay people. Of the five surveyed groups, four found “sickest-first” to be the most important prioritization principle in all three scenarios, and only the physicians group documented a disagreement. In the first scenario, physicians regarded “sickest-first” and “combined-criteria” to be of equal importance. In general, no differences were documented between the examined groups in comparison with lay people in the preference of options in all three scenarios; however, physicians were more likely to choose “combination” in both the second and third scenarios (OR 3.70, 95% CI 1.62–8.44, and 2.62, 95% CI 1.48–4.59; *p* < 0.01), and were less likely to choose “sickest-first” as the single most important prioritization principle (OR 0.57, CI 0.37–0.88, and 0.57; 95% CI 0.36–0.88; *p* < 0.01). Out of 100 free comments, 27 (27.0%) thought that the “social-value” of patients should also be considered, adding the 10th potential allocation principle. Our findings are concordant with literature in terms of allocating scarce medical resources. However, “social-value” appeared as an important principle that should be addressed when prioritizing scarce medical resources in Jordan.

## Introduction

Ethical dilemmas have always been ingrained in the practice of medicine, despite the belief that the right to maximum healthcare should not be compromised (1). However, under certain circumstances, like the shortage of medical resources during crises, when the demand for healthcare services exceeds the supply, this right might be waived (2–4). Pandemics, conflicts and war, and natural disasters are all settings where medical resources can become scarce, posing several challenges (5), albeit resources can be scarce and the decisions to prioritize them can still be faced in daily practice in most health systems. These challenges often leave healthcare providers in conundrums they cannot solve without jeopardizing their commitment to an ethical framework of fairness, equity, and equality (6, 7).

This particular encounter has become an imminent reality during the COVID-19 pandemic. The higher mortality rates for older patients and limited hospital beds and ventilators, in addition to the shortage and exhaustion of healthcare workers left physicians facing tough decisions (8). Multiple studies attempted to alleviate this burden by the construction of an ethical framework for prioritizing patients in the setting of resource scarcity. Other studies have developed ethical approaches for evaluating healthcare decisions in a priority-setting, and proposed criteria and guidelines to direct the fair allocation of the scarce medical resources (9–14). Questions targeting the allocation of ventilators and ICU beds are examples that have been reiterated in literature (15–18). Nine ethical principles are often used to stratify patients in order of priority; sickest first, waiting list, prognosis, youngest first, instrumental values, lottery, monetary contribution, reciprocity, and individual behavior (14, 15). A few presented the perception of healthcare workers and the general public on this topic, and whether individual characteristics should be taken into consideration as part of the decision-making process (19–24).

In a study of 1,267 participants responding to an online questionnaire in which they were asked to prioritize patients in 3 limited-resource settings: scarce donor organs, hospital beds during an epidemic, and joint replacements (19); lay people believed that the “sickest-first” (95% CI 81.2–86.2%) and “first-come, first served” (95% CI 66.2–72.4%) were of top priority. On the other hand, both general practitioners and medical students believed that patients should be ranked based on prognosis (95% CI 74.2–84.9%), or a combination of criteria (95% CI 66.4–78.5%) leaving the degree of sickness as their third priority option. Interestingly, “lottery,” “reciprocity,” “instrumental value,” and “monetary contribution” were considered unfair principles by both groups.

In another study, the opinions of Jewish religious scholars were inconsistent. They varied between leaving the decision to chance—based on the belief that only God decides people's fate, lottery, or first come, first served, and delegating the decision to ethical committees (25). When ~500 Canadian participants were surveyed, over 90% of respondents agreed that the most important goal of pandemic preparations was saving lives. Individuals of older age (OR = 8.51, *p* < 0.05) and employment (OR = 9.48, *p* < 0.05) were agreed to be of highest priority (26). Furthermore, an article comparing the public community and local authorities in Australia reported similar views in both groups; healthcare workers should be prioritized, followed by viral and vaccine researchers and developers (27) in support of the “instrumental-value” principle. Treating the young was considered more ethical, but the elderly believed that patients' overall well-being should affect prioritization, rather than age (28). Scarcity of organs for donation serves as another example where ethical dilemma might ensue. Priority should be given to maximize benefit which respondents believed meant targeting younger patients or those who have a worse prognosis. Waiting list was considered of lower priority, as were individuals who engaged in socially undesirable behaviors, especially if they were liable for their illness (29–31).

Jordan is a lower-middle income country (32) with a population of 9.9 million (33) most of whom reside in the capital, Amman. More than 94% of Jordanians are Muslims and approximately 6% are Christians (34). In 2019, the Gross Domestic Product (GDP) in Jordan was worth 43.74 billion US dollars (35). Unemployment rate in 2019 was 14.7% (36), and 31.9% of Jordanians were not covered by health insurance (37). The health care system in Jordan is divided between the private and public sector. With a total of 106 hospitals and 12,081 beds, the public sector accounts for 67% of beds. Up until 2017, Jordan had 2.3 physicians and 2.8 nurses per 1,000 people (38). Even though Jordan is known for its high-quality healthcare services, both the escalating population growth rate and the recent increase in refugee numbers render the current bed availability in Jordan deficient (39).

The objective of our study is to explore the moral intuitions held by the different members of the Jordanian society (religion scholars, physicians, medical students, allied health practitioners, and lay people) on several topics that have arisen in light of limited medical resources. It also aims to explore whether or not different participant groups of the same society will have different perceptions on the way resources should be allocated and the way their results will compared to that of international literature.

## Methodology

An online survey containing three hypothetical scenarios of scarcity of medical resources including organ donation, hospital beds amid flu epidemics, and novel therapeutics for lung cancer patients was distributed. The first two scenarios along with the allocation criteria were developed in a previous study by Krütli et al. (19), permission was sought from the corresponding authors. The third scenario, however, was new and addressed the allocation of expensive and novel therapeutic drugs for cancer patients. All scenarios were initially written in English and then translated to Arabic, validated then piloted and modified accordingly. Identical English and Arabic versions of the scenarios were shared with participants allowing them to choose their preferred language. The survey asked participants to rank the allocation criteria for fair distribution of certain limited resources from the most important (score-1) to the least important (score-9) prioritizing principle. In addition, a free-comment text option was allowed at the end of the survey.

The following principles and their definitions were used,

- Behavior: priority to those who have not become ill by own fault.
- Instrumental value: priority to those who have essential roles for keeping society operational (e.g., hospital staff).
- Monetary: substantial contribution to the costs of the treatment.
- Order: according to the order of registration.
- Random: random selection, e.g., via a lottery.
- Service: contribution in the past to the common good (e.g., by volunteering).
- Sickest first: the sickest individuals to be given priority.
- Survival: the likelihood to survive the longest.
- Youngest: prioritizing young individuals.
- Combination: a combination of criteria including age (youngest first), and prognosis (longest survival with intervention).

In the first scenario: a team of medical consultants was responsible for allocating 100 kidneys from eligible donors. However, 500 individuals needed a kidney transplant. For convenience, we assumed that all 500 individuals were eligible for the transplant. The following allocation principles were used in this order: sickest, order, survival, behavior, young, random, combined, service, and money. In the second scenario: a very severe flu epidemic hit a mid-sized town of ~50,000 inhabitants. There were, however, only 500 hospital beds available and 2,500 individuals who needed hospital care. The following allocation principles were used in this order: sickest, order, survival, instrumental value, combined, young, random, service, and money. In the third scenario: One hundred lung cancer patients were tested for a novel targeted treatment that will cost 10,000 JDs (14,000 USD) per patient. The financial coverage was available for ten patients. For convenience, all 100 patients were assumed eligible for the treatment. The following allocation principles were used in this order: sickest, order, survival, behavior, young, random, combined, service, and money

The research protocol was approved by the Institutional Review Board (IRB) at King Hussein Cancer Center (KHCC). Informed consent documentation was waived, and a cover page that informed all participants about the purpose of this study was used. Data collection took place between the 27th of April 2020 and the 18th of May 2020. The research team aimed to target five groups of individuals: religion scholars, physicians, medical students, allied health practitioners and lay people. The objective was to enable a comparison between the various groups. This was achieved through sending an email with the questionnaire's link to the staff at several healthcare facilities in order to reach to professionals (physicians, allied health professionals including nurses, and pharmacists). Medical students were targeted through many of the co-authors, as well as contacting deans of the medical schools in Jordan to share the link with the students. Lay people were targeted through social media channels and through the snowball effect where those who completed the questionnaire were asked to share the link with friends and relatives. Religion scholars were identified and communicated with by one of the authors (AM). Of those, participants 18 years and above were then selected.

Descriptive analyses including the mean, median, frequency, and percentages were used to describe the numerical and categorical demographic data of the participants, as well as their preferred prioritization principle. Odds ratio extracted out of the logistic regression was reported with the corresponding 95% confidence interval (CI), and was used to compare opinions amongst all groups in comparison to the lay people group, which served as a reference. Additionally, gender was taken into consideration, where male vs. female was tested among the whole sample with a specific comparison among physicians. A significant *p* ≤ 0.05 was used as the cut-off.

## Results

### The Whole Group

A total of 1,286 survey responses were gathered, out of which 58.6% (*n* = 754) of the respondents completed at least one scenario. There were no significant gender-based or age-based differences between those who completed the survey and those who did not (*p* = 0.328, and 0.860, respectively). The mean and median age for all participants was 35.5 and 33 years, respectively, with an age range of 18–78 years. There were 372 males (49.3%), and 382 females (50.7%). The majority had an undergraduate degree (*n* = 469, 62.2%). Table 1 details the demographics of the participating groups.

**Table 1:** Demographics of the study group (*N* = 754).

	***n* (%)**	**Total 754 (100)**	**Religion scholars 30 (3.9)**	**Physicians 166 (22)**	**Medical students 162 (21.5)**	**Allied health 122 (16.2)**	**Lay people 274 (36.3)**
Gender	Male	372 (49.3)	24 (80)	99 (59.6)	73 (45.1)	36 (29.5)	140 (51.1)
	Female	382 (50.7)	6 (20)	67 (40.4)	89 (54.9)	86 (70.5)	134 (48.9)
Educational level	Primary	47 (6.2)	3 (10)	0 (0)	0 (0)	2 (1.6)	13 (4.7)
	Undergraduate education	469 (62.2)	9 (30)	48 (28.9)	158 (97.5)	84 (68.9)	199 (72.6)
	Higher Education	238 (31.6)	18 (60)	118 (71.1)	4 (2.5)	36 (29.5)	62 (22.6)
Age (years)	Min	18	29	22	18	20	19
	Max	78	70	78	26	62	75
	Mean	35.5	48	43.1	21.5	32.6	38.7
	Median	32	46.5	45	22	29.5	37

### Detailed Data on Subgroups

**Religion Scholars:** There were 30 (3.9%) participants, with predominance of males (*n* = 24, 80.0%) and a mean age of 48 years. The majority (*n* = 18, 60.0%) completed postgraduate studies.

**Physicians:** There were 166 (22.0%) participants, with predominance of males (*n* = 99, 59.6%) and a mean age of 43 years. The majority of physicians completed postgraduate studies (*n* = 118, 71.1%) and practiced medicine in Jordan (*n* = 115, 70.1%), followed by Arab countries (*n* = 32, 19.5%), and 17 (10.4%) practiced in Western countries.

**Medical Students:** There were 162 (21.5%) participants, with a slight predominance of females (*n* = 89, 54.9%) and a mean age of 21.5 years. The vast majority had undergraduate education (*n* = 158, 97.5%).

**Allied Health Practitioners:** There were 122 (16.2%) participants, with a predominance of females (*n* = 86, 70.5%) and a mean age of 32.6 years. The majority (*n* = 84, 68.9%) with undergraduate studies.

**Lay People:** This constituted the largest group, with a total of 274 (36.3%) respondents. There was an almost equal representation of both genders (males *n* = 140, 51.1%, and females *n* = 134, 49.9%). The mean age of the group was 38.7 years. Most had undergraduate studies (*n* = 199, 72.6%).

### Prioritization Principle Allocation

Overall, the most commonly prioritized principle was “sickest first” in all 3 scenarios, except for physicians in the first scenario where “sickest first” and “combination” were of equal importance. In the first scenario, the second most common prioritization principle chosen by all groups was the “combination.” For the second scenario the second most chosen principle was “survival” in all groups. For the third scenario the second most chosen allocation principle was “survival” in all groups except for physicians and medical students who chose “combination” as their second prioritization principle. Interestingly, “monetary,” and “service” were the least favored principles in all scenarios among all groups (Table 2 demonstrates the 3 scenarios with scoring of the priority principles among all groups).

**Table 2:** Percentages of respondents who chose each allocation principle as the most important one among the study group.

**Percentage (%)**	**Sickest First**	**Order**	**Survival**	**Behavior**	**Young first**	**Random**	**Combination**	**Service**	**Monetary**	***N***
**Scenario 1. Organ donation for transplant**										
Religion scholars	60.0	10.0	6.7	3.3	3.3	3.3	13.3	0	0	30
Physicians	33.1	11.4	14.5	3.0	3.6	0.6	33.1	0.6	0	166
Medical Students	48.8	7.4	14.2	1.9	1.2	1.2	24.7	0.6	0	162
Allied Health	51.6	7.4	11.5	4.9	2.5	0	22.1	0	0	122
Lay people	55.1	9.5	12.0	1.8	2.2	2.2	16.4	0.4	0.4	274
Total	48.5	9.2	12.7	2.7	2.4	1.3	22.7	0.4	0.1	754
**Scenario 2. Flu epidemic**										
Religious Leaders	54.2	0	25.0	20.8	0	0	0	0	0	24
Physician	44.5	3.6	20.4	8.8	13.9	0	7.3	0.7	0.7	137
Medical Students	53.3	6.6	16.7	4.4	12.4	1.5	4.4	0.7	0	137
Allied Health	59.0	2.0	12.0	9.0	7.0	1.0	10.0	0	0	100
Lay people	58.3	6.9	12.9	9.3	4.2	1.4	6.9	0.0	0	216
Total	54.1	5.0	15.8	8.5	8.5	1.0	6.7	0.3	0.2	614
**Scenario 3. Expensive cancer medication**										
Religious Leaders	60.9	13	13.0	0	4.3	0	4.3	0	4.3	23
Physician	41.2	5.1	21.3	3.7	0.7	0	27.2	0	0.7	136
Medical Students	55.2	7.5	16.4	1.5	0	1.5	17.2	0	0.7	134
Allied Health	56.8	6.3	15.8	4.2	1.1	1.1	12.6	0	2.1	95
Lay people	55.0	8.0	17.5	2.0	1.5	2.0	12.5	0.0	1.5	200
Total	52.4	7.1	17.7	2.6	1.0	1.2	16.7	0.5	1.4	588

#### Scenario One

This was answered by 754 participants (100.0%). In general, all groups concurred with “sickest first” as the main allocation principle chosen by 48.5%, apart from physicians. In second place, the “combination” principle was chosen by all groups. In detail, 60% of religion scholars chose the “sickest first” as the mainstay principle to allocate resources, followed by lay people where 55% chose it as the priority principle. The allied health practitioners chose sickest first in 51.6% of responses, physicians, however, chose “sickest first “and “combination” principle as equal priority principles. Medical students' first option was “sickest first” in 48.8% of responses. It is worth noting that the contribution to financial cost “monetary,” and voluntary contribution “services” were the least selected principles among all groups.

#### Scenario Two

This was answered by 614 participants (81.0%). The overall most commonly chosen principle was “sickest first” (54.1%). This was chosen by 59.0% of allied health practioners, followed by 58.3% of lay people, 54.2% of religion scholars, 53.3% of medical students, and 44.5% of physicians. The second most common choice for all groups was “survival.” The least commonly chosen principle was the contribution to cost of treatment “monetary” and “services.” It is worth noting that the “instrumental value” was favored twice as much by religion scholars in comparison to other groups.

#### Scenario Three

This was answered by 588 participants (78.0%). This scenario was especially designed to address the special needs of cancer patients in the era of personalized medicine and the cost of novel medication. Overall, the most commonly chosen principle was “sickest first” (52.4%). This was chosen by 60.9% of religion scholars, 56.8% of the allied health professionals, 55.2% of medical students, 55.0% of lay people, and lastly, 41.2% of physicians. The “combination” principle was chosen by 27.2% of the physicians. An interesting finding unique to this scenario is that in comparison to other scenarios, the contribution to cost “monetary” principle was chosen by a larger percentage of participants. The least commonly chosen principle remained to be “service.”

### Comparison Between all Groups With Reference to Lay People

Overall, no differences were noted when comparing religion scholars and allied health practitioners to lay people in the preference of options for all scenarios. However, differences between physicians' and lay people's prioritization principles were noted (Supplementary Table 1). When compared to lay people, physicians were less likely to choose “sickest first” as their top priority (OR 0.46; 95% CI 0.31–0.69; *p* < 0.01) in the first scenario. Physicians ranked “sickest first” and “combination” as equally important in priority to allocate scarce medical resources (OR 2.52; 95% CI 1.60–3.97; *p* < 0.01). In the second scenario, physicians tended to choose the “survival” as the principle to allocate the scarce medical resources (OR 1.72; 95% CI 0.97–3.06; *p* = 0.06). Physicians were more likely to choose “combination” in the second and third scenarios (OR 3.70, 95% CI 1.62–8.44, and 2.62, 95% CI 1.48–4.59; *p* < 0.01). Physicians were less likely to choose the “sickest first” option as the single most important priority principle (OR 0.57, CI 0.37–0.88, and 0.57; 95% CI 0.36–0.88; *p* = 0.01), in comparison to lay people.

Medical students were more likely to choose the “combination” as their top priority in the first and second scenarios when compared to lay people (OR 1.67, 3.26; 95% CI 1.03–2.69, and 1.40–7.53; *p* = 0.04, and 0.01, respectively).

### Comparisons Based on Gender Among the Different Scenarios

Males were more likely to choose “random” (OR 1.97; 95% CI = 1.02–3.80, *p* = 0.04) in the second scenario, and “combination” (OR 1.57; 95% CI = 1.01–2.43, *p* = 0.04) in the third scenario, in comparison to females. However, gender was not a significant factor to stratify the preferences among physicians.

### The Free Text Comments Analysis

One hundred (13.3%) participants added free comments that addressed their opinion. Each comment was then stratified based on one of the three principles of ethics: autonomy (*n* = 8), beneficence (*n* = 30) and justice (*n* = 60). In two comments, the link to any of the principles could not be determined. Among the comments, 27 (27.0%) thought that the “social-value” of patients, i.e., being the principle care- and food providers to the family, should be considered.

## Discussion

Overall, our results clearly indicate that “sickest first” is the prioritization principle that should be considered when encountering scarce medical resources in all three scenarios. In general, there was an overall concordance between participants from the five different groups.

Throughout history, physicians have been faced with the difficult decision of prioritizing patients amid scarcity of essential medical resources. Currently, physicians are forced to decide on the allocation of intensive care unit beds and ventilators in overwhelmed facilities dealing with SARS-CoV-2 infection (18, 40). In countries where the economy is poor, this scenario tends to recur often (41, 42).

We carefully chose to discuss three particular scarce resources. The first scenario, organ donation for transplantation, was chosen as a universal dilemma; there will always be less organs available than there are patients on the waiting lists for the foreseeable future. In Jordan and other countries in the region, this is a particularly scarce resource, not only due to limitations in facilities and trained personnel, but also because there is still concern regarding organ donation (43). The second scenario addresses the shortage of hospital beds during a flu epidemic. This is analogous to the current situation in light of the COVID-19 pandemic (44), which has resulted in a plethora of publications and discussions on this particular issue (15–18, 25). The third scenario aimed to address the limitation to the availability of expensive novel therapeutics to cancer patients, including targeted therapies and immunotherapy. The costs of the novel drugs are exhausting the medical sector in countries with limited resources, further widening the gap between cancer patients worldwide. Other examples of resources that could become at some point scarce are ventilators, medical staff, and vaccines (45).

There are nine common ethical principles, and a multitude of varying opinions on how to rank them according to priority (14, 45). It would be a huge relief to decision-makers, however, if there was a clear consensus regarding how to allocate scarce medical resources. The criteria for patient selection and the allocation of resources should be transparent, yet a clear-cut approach to the development of such guidelines might not be easily attained. The trend across various studies regarding the allocation of organs to those on waiting lists is to prioritize maximizing benefit while attempting to achieve equity (46, 47). In light of the COVID-19 pandemic, many articles aimed to set guidelines regarding the rationing of scarce healthcare resources during this crisis (40).

The quality-adjusted life-year (QALY) is a measure of the years of life remaining for a patient following a particular treatment or intervention. By including both the quality and the quantity of life lived, QALY became a favored tool in healthcare priority settings (48). Patients with the lowest cost per QALY are usually prioritized in scarce medical resource allocation, therefore increasing health benefit and social welfare (49). One popular study argues that the value of maximizing benefits is the most fundamental in prioritizing patients, including saving the most lives as well as saving the most life-years—thus maximize prognosis (15). However, QALY and health-benefit maximization are so often criticized for having the potential to be “ageist” because life expectancy is part of QALY calculation. Elderly, with a shorter life expectancy will be given the lowest cost per QALY and are therefore the least prioritized (50).

Another way of prioritization is explored by Golan et al study (51), which demonstrated a conjoint analysis method (also known as discrete choice experiments) which aimed to derive weights for a set of criteria related primarily to “benefits from technologies.” Weights for criteria were measured by an internet-based software as respondents were asked 40 questions about choosing between two hypothetical technologies which were defined in terms of just two criteria, whereby one of the technologies had a higher performance rating on one criterion and a lower rating on the other criterion than the other technology. So when answering, respondents had to make a tradeoff and a choice. The advantage authors saw in this method relative to alternative scaling methods used in our survey, was that choice is natural and people, knowingly or unknowingly, experience similar situations daily. Our study results showed that among the three scenarios, “the-sickest” was the most important priority principle, where in this study the most important criterion was “lives-saved and statistical lives” with similar weight to “quality-of-life gains” and “life-prolongation benefits,” all of which were related to the principle of “need,” defined as the extent to which a technology is expected to achieve any of the ultimate health goals of saving and prolonging life and or improving health-related quality of life (HRQoL).

Multicriteria decision analysis (MCDA) is yet another frequently used method in literature to make decisions for prioritizing alternatives that are ranked based on a variety of criteria (52, 53). In a pilot study conducted in New Zeeland (54), the authors conducted a discrete choice experiment. The survey was conducted using 1000 Minds software (55), which asks participants to choose between hypothetical patients who could be treated by the healthcare technologies. It used the potentially all pairwise rankings of all possible alternatives (PAPRIKA) method (56), which identifies all pairs of hypothetical patients defined on two criteria at a time that involve a trade-off. Each participant was asked to rank pairs of patients and eliminated pairs that can be identified by transitivity. For example, if a participant prioritizes patient A over patient B, and then patient B over patient C, then patient A is prioritized over patient C by transitivity and the software will not ask the participant to rank the third pair of patients. At the end, six benefit-related criteria were created.

An ongoing question is who gets to decide these guidelines? In other words, who gets to decide who lives? (57) Many people may intuitively say that this burden falls in the hands of physicians; while others believe that all members of society should be involved (58, 59). We decided to explore the opinions of five groups, with the goal of determining the collective-group opinions and comparing the results to explore any significant differences. We included lay people, since their values might diverge with those of physicians (59). Our findings clearly indicate that there are no major differences in opinion regarding the allocation of scarce resources in the three scenarios. All groups in our study considered the “sickest-first” principle as the most important allocation principle in the 3 hypothetical scenarios, while “monetary contribution” and “reciprocity” were found to be the least important. This is similar to the study by Krütli et al. (19), in which the most important allocation priorities for lay people were “sickest-first” and “waiting-list,” whereas “lottery,” “monetary contribution” and “reciprocity” received the lowest rank and were considered unfair. Physicians were more likely to choose “prognosis,” “combined criteria,” and “youngest first” in all 3 hypothetical scenarios but were less likely to choose “waiting-list” and “sickest-first” except in the allocation of joint replacement surgery.

An ethicist's perception on how scarce medical resources should be allocated might provide a reasonable source of prioritization. In two studies conducted by Persad et al. (14) and Emanuel et al. (15), ethicists prioritized maximizing the total benefit which includes “saving more lives” and “life-years saved” or prognosis. All other principles were used to facilitate decision making when two patients have an equal prognoses. They considered “sickest-first” and “waiting list” as morally unacceptable. In Jordan, the ethicist's role is still emerging. However, similar to other countries in the region, religion scholars play a major role in contemplating issues of everyday life and are viewed by many to hold the most ethical and just decisions based on the creed. For example, during the recent COVID-19 outbreak, the Jordanian government recommended the closure of mosques and churches as part of their social-distancing measures. This unfavorable decision was frowned upon by a large number of the lay people who refused to comply until Muslim and Christian scholars alike publicly stated their support of the decision as it represents what is best for society (60).

We do not presume our findings are the solution to the aforementioned ethical dilemmas, albeit we believe that empirical research into these attitudes can be useful in many ways. By showing which beliefs are most adopted by the public, and which are commonly regarded as frank, physicians can make their informed decisions when faced with scenarios of limited resources. Persad wrote “even though popularity does not constitute correctness, the unpopularity of a normative position can justify placing it under scrutiny.” (45).

We have attempted to address participants' concerns, comments, and other ideas that could have evaded inclusion among the nine ethical principles. The “social-value” of individuals was presented as an additional ethical principle that was not previously included. This is defined as the presence of social- and financial-liability on the patients, such as children, elderly parents, or siblings, so that his/ her loss cannot be compensated. In the absence of well-developed national security system in countries like Jordan to support dependent individuals, especially elderly parents and young offspring, those individuals might find themselves in jeopardy if their primary caregiver is lost.

Interestingly, voice messages were sent from some of participants to the corresponding author on the overwhelming feelings they experienced while completing the survey. They found it “morally draining” once they imagined themselves in a position to take decisions to prioritize the scarce resources or as patients awaiting the decision to be made by others on whether or not they will be prioritized (Personal communication)

## Limitations

We acknowledge limitations in our study. Some participants completed only one or two of the scenarios, but their responses were still included in the study. This could be attributed to the emotional burden that comes with being faced with choices that all seem rational to allocate scarce medical resources. This is especially critical in times of the COVID-19 pandemic. Moreover, the choices in each of the three scenarios were put in the same order without randomization, possibly creating a raw-effect bias which might have contributed to participants selecting the “sickest-first” option more often. However, this is the first attempt to delve into this repressing exercise of trying to allocate the scarce medical resources within our population.

## Conclusion

In conclusion, our findings are at large consistent with international literature in terms of prioritizing patients under conditions of scarce medical resources. In addition, “social-value” appeared to be an important priority principle, most likely unique to the region, where social security systems are under-developed. We recommend considering the findings in our study by policymakers when allocation of scarce medical resources is an issue, such as with the COVID-19 pandemic. Repeating the study after the pandemic should be considered, the results might vary given that the participants would have been subjected to a real-life example.

## Data Availability Statement

The raw data supporting the conclusions of this article will be made available by the authors, without undue reservation.

## Ethics Statement

The studies involving human participants were reviewed and approved by IRB ethics committee at King Hussein Cancer Center. Written informed consent for participation was not required for this study in accordance with the national legislation and the institutional requirements.

## Author Contributions

AMas and MA-H inception of the idea, critical review of the first draft, critical review, and final approval. MY and YA-S collection of data, writing the first draft, review, and final approval. RM literature review, reviewing and editing the first draft, final review, and approval. HS, AMan, YA, NA, and RS collection of data, reviewing and editing the first draft, final review, and approval. All authors are accountable for the content of the manuscript.

## Conflict of Interest

The authors declare that the research was conducted in the absence of any commercial or financial relationships that could be construed as a potential conflict of interest.
