# Supplementary material for: The Fair Allocation of Scarce Medical Resources: A Comparative Study From Jordan
Source: Front Med (Lausanne). 2021 Jan 12;7:603406. doi: 10.3389/fmed.2020.603406 (PMC7873904; doi:10.3389/fmed.2020.603406)
Supplement: Supplementary file 1 [file Table_1.docx]

Table-S1: Odds Ratio for the various groups in comparison to lay people.

| SC | Principle | Religion scholars | 95% CI | Physicians | 95% CI | Medical Students | 95% CI | Allied Health practitioners | 95% CI |
| --- | --- | --- | --- | --- | --- | --- | --- | --- | --- |
| 1 | Sickest First | 1.22 | 0.57- 2.63 | 0.46 | 0.31-0.69* | 0.78 | 0.53-1.14 | 0.87 | 0.57- 1.33 |
|  | Order | 1.06 | 0.30-3.73 | 1.23 | 0.65-2.30 | 0.76 | 0.37- 1.55 | 0.76 | 0.34- 1.67 |
|  | Survival | 0.52 | 0.11- 2.29 | 1.23 | 0.70- 2.17 | 1.21 | 0.68- 2.14 | 0.95 | 0.48- 1.84 |
|  | Behavior | 1.86 | 0.20- 16.42 | 1.67 | 0.47- 5.86 | 1.02 | 0.23- 4.30 | 2.78 | 0.83- 9.30 |
|  | Young First | 1.54 | 0.17- 13.24 | 1.68 | 0.53- 5.28 | 0.56 | 0.11- 2.79 | 1.13 | 0.27- 4.57 |
|  | Random | 1.54 | 0.17- 13.24 | 0.27 | 0.03- 2.26 | 0.56 | 0.11- 2.79 | 0.17 | 0.00- 3.01 |
|  | Combination | 0.78 | 0.26- 2.35 | 2.52 | 1.60- 3.97* | 1.67 | 1.03- 2.69** | 1.45 | 0.84-2.46 |
|  | Service | 2.99 | 0.11-74.99 | 1.65 | 0.10- 26.63 | 1.70 | 0.10- 27.29 | 0.74 | 0.03- 18.39 |
|  | Monetary | 2.99 | 0.11- 74.99 | 0.55 | 0.02- 13.51 | 0.56 | 0.02- 13.85 | 0.74 | 0.03- 18.39 |
| 2 | Sickest First | 0.84 | 0.36-1.96 | 0.57 | 0.37-0.88 | 0.81 | 0.52- 1.25 | 1.03 | 0.63-1.66 |
|  | Order | 0.27 | 0.01-4.57 | 0.51 | 0.18- 1.42 | 0.94 | 0.40-2.21 | 0.27 | 0.06- 1.21 |
|  | Survival | 2.24 | 0.81-6.11 | 1.72 | 0.97- 3.06 | 1.35 | 0.74- 2.46 | 0.92 | 0.44-1.88 |
|  | Behavior | 2.58 | 0.86- 7.64 | 0.94 | 0.44- 1.99 | 0.45 | 0.17- 1.14 | 0.97 | 0.42- 2.21 |
|  | Combination | 0.45 | 0.02- 7.89 | 3.70 | 1.62- 8.44* | 3.26 | 1.40- 7.53* | 1.73 | 0.62- 4.78 |
|  | Young first | 1.24 | 0.06- 24.81 | 0.22 | 0.01- 4.32 | 1.05 | 0.17- 6.37 | 0.72 | 0.07- 6.98 |
|  | Random | 0.27 | 0.01- 4.57 | 1.06 | 0.45- 2.42 | 0.61 | 0.23- 1.62 | 1.49 | 0.64- 3.44 |
|  | Service | 8.84 | 0.17- 455,37 | 4.76 | .019-117.64 | 4.76 | 0.19-117.64 | 2.15 | 0.04- 109.35 |
|  | Monetary | 8.84 | 0.17- 455.37 | 4.76 | .19- 117.64 | 1.57 | 0.03- 79.82 | 2.15 | 0.04- 109.35 |
| 3 | Sickest First | 1.27 | 0.52-3.07 | 0.57 | 0.36-0.88*** | 1.01 | 0.64-1.56 | 1.08 | 0.65-1.76 |
|  | Order | 1.73 | 0.46-6.43 | 0.62 | 0.24-01.56 | 0.93 | 0.40-2.11 | 0.78 | 0.29-2.04 |
|  | Survival | 0.71 | 0.19-2.51 | 1.28 | 0.73- 2.21 | 0.93 | 0.51- 1.66 | 0.88 | 0.45- 1.71 |
|  | Behavior | 0.93 | 0.04- 17.80 | 1.87 | 0.49- 7.09 | 0.89 | 0.16- 4.94 | 2.15 | 0.52-8.80 |
|  | Young First | 2.98 | 0.29- 29.94 | 0.49 | 0.05- 4.72 | 0.25 | 0.01- 4.89 | 0.70 | 0.07- 6.80 |
|  | Random | 0.93 | 0.04- 17.80 | 0.16 | 0.00- 2.99 | 0.74 | 0.13- 4.11 | 0.52 | 0.05-4.72 |
|  | Combination | 0.32 | 0.04- 2.46 | 2.62 | 1.48- 4.59* | 1.45 | 0.78- 2.68 | 1.01 | 0.48-2.11 |
|  | Service | 8.53 | 0.16- 440.11 | 1.47 | 0.02- 74.47 | 1.49 | 0.02- 75.56 | 2.10 | 0.04-106.61 |
|  | Monetary | 2.98 | 0.29- 29.94 | 0.49 | 0.05- 4.72 | 4937.00 | 0.05- 4.79 | 1.41 | 0.23-8.59 |
|  | Abbreviations: CI, Confidence Interval; SC, Scenarios.  *p < 0.01  **p = 0.04  ***p = 0.01 | | | | | | | | |
